# Drug delivery systems for oral disease applications

**DOI:** 10.1590/1678-7757-2021-0349

**Published:** 2022-02-09

**Authors:** Yue Zhang, Ruining Jiang, Lei Lei, Yingming Yang, Tao Hu

**Affiliations:** 1 Sichuan University West China Hospital of Stomatology Department of Preventive Dentistry Chengdu China Sichuan University, West China Hospital of Stomatology, Department of Preventive Dentistry, State Key Laboratory of Oral Diseases, Chengdu, China.

**Keywords:** Delivery systems, Dental caries, Hydrogels, Nanoparticles, Periodontal disease

## Abstract

There are many restrictions on topical medications for the oral cavity. Various factors affect the topical application of drugs in the oral cavity, an open and complex environment. The complex physical and chemical environment of the oral cavity, such as saliva and food, will influence the effect of free drugs. Therefore, drug delivery systems have served as supporting structures or as carriers loading active ingredients, such as antimicrobial agents and growth factors (GFs), to promote antibacterial properties, tissue regeneration, and engineering for drug diffusion. These drug delivery systems are considered in the prevention and treatment of dental caries, periodontal disease, periapical disease, the delivery of anesthetic drugs, etc. These carrier materials are designed in different ways for clinical application, including nanoparticles, hydrogels, nanofibers, films, and scaffolds. This review aimed to summarize the advantages and disadvantages of different carrier materials. We discuss synthesis methods and their application scope to provide new perspectives for the development and preparation of more favorable and effective local oral drug delivery systems.

## Introduction

The oral cavity is a complex environment which communicates with the external environment, the upper respiratory tract, and the digestive system.^1,2^ A wide variety of microorganisms is present in the oral cavity, including bacteria and fungi, such as *Streptococcus mutans (S. mutans), Lactobacillus spp. Porphyromonas gingivalis (P. gingivalis)*, and *Candida albicans.*^3–5^ These microorganisms colonize different parts of the oral cavity, including tooth surfaces and the periodontium, and form biofilms which invade oral cavity tissues. Periodontitis is a consequence of alterations in the ecology of resident microbial communities. The intimate interaction of bacteria with the host leads to an inflammatory reaction.^6^

Dental caries is a consequence of dietary, sugar-driven biofilm accumulation and localized acidification. Due to frequent sugar consumption, the development of a predominantly acidic environment will favor the growth of aciduric bacteria in the biofilm. Aciduric bacteria include S*treptococcus spp., Lactobacillus spp.*, etc. As a result, the dynamic balance between commensals and opportunistic pathogens is disrupted, causing deleterious microbial community shifts and disrupting tooth-enamel mineral homeostasis.^7–10^

According to the global burden of disease (GBD) study, permanent tooth caries is among the ten diseases with the highest incidence for years lived with disability.^11^ Periodontal diseases comprise a wide range of inflammatory conditions affecting tooth-supporting structures (the gingiva, bone, and periodontal ligaments), which starts with the localized inflammation of the gingiva, initiated by a microbial biofilm that forms on the teeth and gingiva.^12–14^ Periodontal diseases lead to teeth loss and contribute to systemic inflammation, which is highly prevalent worldwide.^15^ Thus, periodontal diseases represent a significant public health problem.^16^ Regarding the topical application of drugs to the oral cavity, the influence of the oral environment on the drugs should be considered. Its complex physical and chemical environment and the complexity of bacterial biofilm affect drug application via the oral cavity.^17^ For example, salivary flow rates in the mouth might affect the efficacy of topical anesthesia to some extent, whereas side effects and drug resistance are inevitable in systemic administration.^18^ Salivary clearance might dilute and weaken the active ingredients in the oral environment. Therefore, carrier systems had to be designed to consistently release active ingredients so their estimated concentrations can be effective. Recently, emerging advanced biomaterials, including hydrogels, films, nanofibers, and particles hold great potential as cell/drug carriers for local drug delivery and biomimetic scaffolds.^19^ Biofilms decrease the effects of drugs on microorganisms, and antibacterial substances are easily metabolized. Moreover, there is no perfect way of delivering drugs to sites such as the periodontal pocket and the periapical area. Drug delivery systems have many advantages, such as increasing drug solubility, prolonging drug action time, improving drug targeting, and reducing cytotoxicity. Moreover, delivery systems play a role in inhibiting bacteria by releasing active ingredients, such as chlorine, and can serve as engineering scaffolds to promote tissue regeneration. For example, patients receiving ibuprofen-incorporated, chitosan-based microspheres show better anti-inflammatory properties than the orally administered ibuprofen group.^20^ Moreover, bone morphogenetic protein 2 (BMP-2) loaded into calcium silicate scaffolds showed continuous release and better stimulation for the induction of mesenchymal stem cells.^21^ In the field of anesthesia, experiments have also proved that appropriate hybrid nanofilms containing the eutectic mixture of 5% lidocaine-prilocaine (LDC-PLC) show a better anesthetic effect, with higher permeability and no cytotoxicity.^22^

**Figure 1:**
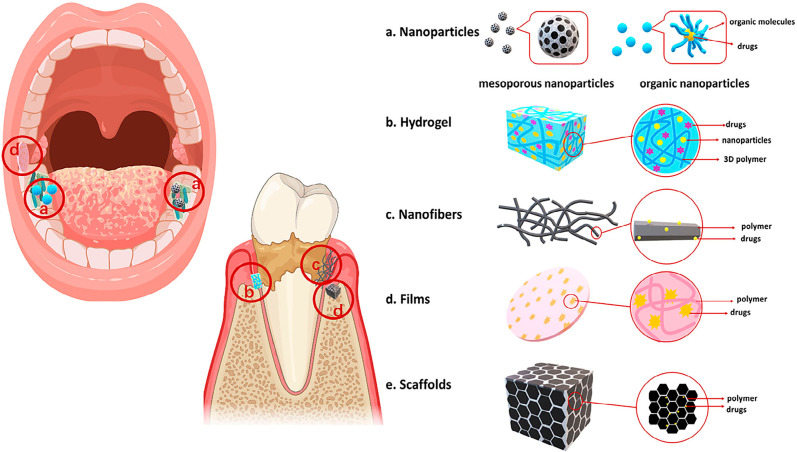
Models of different material forms. a: Nanoparticles include inorganic and organic carriers. As shown in the figure, these are mesoporous nanoparticles. b: Hydrogels are usually made from 3D polymers and can be loaded with drugs or nanoparticles. c: Nanofibers are fibers of approximately 100 nm in diameter which can carry drugs. d: Films are often used for oral mucosal drug delivery. e: Scaffolds are loaded with growth factors and usually used in tissue regeneration

In the reviewing process, “oral/oral cavity/mouth/mouth cavity” and “drug delivery systems/carrier materials/drug targeting” were used as research items in the Pubmed, MEDLINE, and Web of Science databases. Relevant articles with an impact factor greater than five and published after 2015 were included. Moreover, we added some articles about the progress of experiments based on previous classic reviews. All drug delivery systems were classified as nanoparticles, hydrogels, nanofibers, and films according to their material properties. The carrier materials used as oral drug delivery systems in recent years are summarized in Table 1, and their different forms are described in Figure 1. This review discusses drug delivery systems in the oral cavity, which are prepared by various methods to adapt to complex situations. For example, they can be used as support structures to promote regeneration in defective tissues or as drug carriers to release active ingredients which control infection. Moreover, to support the future development of carrier materials, we summarized their composition and application.

**Table 1:** Carrier materials used as oral drug delivery systems in recent years

Drug delivery system		Materials	Active ingredients	Biological activity	Diseases	Experiments	References
Nanoparticles	Inorganic nanoparticles	Amine-functionalized expanded pore mesoporous silica (aMSN)	Polyacrylic acid-stabilized amorphous calcium phosphate (PAA-ACP)	Promote re-mineralization	Enamel white spot lesions	*In vitro*	Hua et al, 2020
		Silver-decorated mesoporous silica nanoparticles (Ag-MSNs)	Chlorhexidine(CHX), Silver nanoparticles	Antibacterial effects	Periodontal diseases	*In vitro; In vivo*	Lu et al, 2018
		Mesoporous calcium silicate nanoparticles (MCSNs)	Gentamicin; Fibroblast growth factor-2 (FGF-2)	Antibacterial effects; Promote tissue regeneration	-	*In vitro*	Huang et al, 2017
			Bone Morphogenetic Protein 2 (BMP-2)	Inducing the differentiation of dental pulp cells and odontoblasts	“Immature permanent tooth with necrotic pulp”	*In vitro*	Huang et al, 2018
		Poly (d,l-lactide-co-glycolide acid) (PLGA)	Chlorhexidine	Antibacterial effects	-	*In vitro*	Priyadarshini et al, 2017
			Grapeseed extract (GSE)	Reduce biodegradability of the dentin collagen matrix	-	*In vitro*	Fawzy etal, 2017
	Organic nanoparticles						
			Lovastatin	Promote dentin differentiation	Dental caries	*In vitro; In vivo*	Lin et al, 2017
		Liposomes	Doxycycline	Antibacterial activity	Periodontal diseases	*In vitro; In vivo*	Hu, F, et al.,2019
		Chitosan	Dexamethasone; Transforming growth factor-β1 (TGF-β1)	Enhance migration, adherence and odontogenic differentiation of stem cells from apical papilla (SCAP)	Apical periodontitis	*In vitro; In vivo*	Shrestha et al, 2019
		O-Carboxymethyl chitosan (OCMCH)	Peptide histatin 5	Antifungal activity	Fungal infections	*In vitro; In vivo*	Park etal, 2017
		Poly(amide-amine) dendrimer (PAMMA)	Apigenin	Antibacterial activity ; Promote remineralization	Dental caries	*In vitro*	Zhu et al, 2018
		Diblock copolymers	Famesol ; Myricetin	Antibacterial activity	Dental caries	*In vitro*	Sims et al, 2020
Hydrogel		Chitosan/β-glycerophosphate (CS/β- GP) thermosensitive hydrogels	Bone morphogenetic protein-7 (BMP-7) ; Ornidazole (ORN)	Promote tissue regeneration; Antibacterial activity	Chronic periodontitis	*In vitro; In vivo*	Zang et al, 2019
			Aspirin; Erythropoietin	Promote tissue regeneration	Periodontitis	*In vitro; In vivo*	Xu etal, 2019
			Naringin	Anti-inflammatory properties	Periodontitis	*In vitro; In vivo*	Chang et al, 2017
		Thermosensitive micellar hydrogel	Ibuprofen ; Basic fibroblast growth factor (bFGF)	Anti-inflammatory activity; Promote tissue regeneration	Peri-implantitis	*In vitro*	Chen et al, 2019
		Hydrogelator Nap-Phe-Phe-Tyr-OH (NapFFY) hydrogels	Stromal cell-derived factor-1 (SDF-1)	Promote tissue regeneration	Periodontal bone destruction	*In vitro; In vivo*	Tan et al, 2019
			Bone morphogenetic proteins (BMPs)		Alveolar bone defects	*In vitro; In vivo*	Pan et al, 2019
		High-stiffness transglutaminase crosslinked gelatins (TG-gels)	IL-4 ; Stromal cell-derived factor (SDF)-1α	Promote tissue regeneration	Periodontitis	*In vitro; In vivo*	He et al, 2019
		Hyaluronic acid (HA) hydrogels	Human dental pulp cells (hDPCs)	Promote tissue regeneration	—	*In vitro; In vivo*	Silva et al, 2018
		Peptide amphiphiles	Ciprofloxacin (CF) and metronidazole (MN);Nitric oxide(NO)	Promote tissue regeneration ; Antibacterial activity	Endodontic infections	*In vitro; In vivo*	Moon et al, 2018
		Nanostructured lipid-biopolymer hydrogel	Lidocaine-prilocaine (LDC-PLC)	Anesthesia	-	*In vitro; In vivo*	Ribeiro et al, 2018
Nanofibers		Polydioxanone nanofibers	Metronidazole (MET); Ciprofloxacin (CIP); Minocycline (MINO)	Antibacterial activity	Periapical disease	*In vitro; In vivo*	Bottino et al, 2019
			Vasajlar endothelial growth factor (VEGF)	Promote tissue regeneration	Immature necrotic teeth	*In vitro; In vivo*	Yadlapati et al, 2017
		Cellulose nanofibers (CNF)	Surfactin	Antibacterial activity		*In vitro*	Johnson et al, 2020
		K-carrageenan oligosaccharides (CO)			Periodontitis		
		Gelatin films	Econazole nitrate (ECN)	Antifungal activity	Stomatitis	*In vitro*	Dolci et al, 2018
		Chitosan; pectin; chitosan-pectin; nanostructured lipid carriers	lidocaine-prilocaine (LDC-PLC)	Anesthesia	-	*In vitro*	Ribeiro et al, 2020
Scaffolds		Polycaprolactone (PCL); polylactic- co-glycolic acid	Adenoviruses	Promote tissue regeneration	Periodontal diseases	*In vitro; In vivo*	Pilipchuk et al, 2018
		Chitosan; alginate; PLGA hybrid scaffolds	Insulin-like growth factor (IGF- 1) and progression factor; Bone morphogenetic factor-6 (BMP-6)	Promote tissue regeneration	Periodontitis	*In vitro*	Duruel.et al, 2017

## Nanoparticles

Nanoparticles have widely served as drug delivery systems in the oral cavity.^23,24^ Nanoparticles include inorganic carriers, such as mesoporous silica and mesoporous calcium silicate and organic carriers, such as poly (d,l-lactide-co-glycolide acid) (PLGA) and chitosan. Nanoparticle size lies mostly between 1-100 nm, which results in good diffusion properties and loading performances.^17,25^ Inorganic nanoparticles are mainly composed of inorganic materials which are crystallized and amorphous solids at ambient temperature. Their reactivity is different in various solution environments, which significantly influence their toxicity. Organic nanoparticles mainly consist of organic substances such as lipids, proteins, etc. Depending on their composition, organic nanoparticles can be degraded and digested in different parts *in vivo*. Organic nanoparticles are generally less toxic than inorganic nanoparticles, but they might cause toxicity under particular circumstances.^26^ In recent years, inorganic nanoparticles have received attention due to their unique materials, which are more inert, stable, and easy to functionalize.^27^

### Inorganic nanoparticles

In general, mesoporous materials are used as inorganic nanoparticle carrier systems. Mesoporous materials include mesoporous silica nanoparticles (MSNs) and mesoporous calcium silicate nanoparticles (MCSNs).^28–30^

MSNs have been modified by amination to make them more suitable for use in the oral environment. In recent studies, the amine-functionalized expanded pore mesoporous silica (aMSN) was synthesized to load polyacrylic acid–stabilized amorphous calcium phosphate (PAA-ACP). PAA-ACP loaded aMSN has the potential to treat white enamel spot lesions and serve as filler in resin adhesives.^31,32^ Regarding their antimicrobial properties, Lu, et al.^33^ (2018). incorporated silver ions into MSNs, synthesizing silver-decorated mesoporous silica nanoparticles (Ag-MSNs) and using them to load chlorhexidine (CHX). They showed redox/pH-responsive release properties due to CHX and silver ions which could inhibit *S. mutans* biofilm growth. Ag-MSNs^@^CHX were more effective than an equivalent amount of free CHX in limiting *S. mutans* biofilm formation since they induced bacterial cell death, particularly in the long term. Though *S. mutans* was only used as a model to evaluate the effect of Ag-MSNs^@^CHX, the material may be considered for treating diseases caused by bacterial biofilms, such as periodontal diseases.

A practical antibacterial component, silver ions were also loaded into mesoporous calcium silicate to prepare nano-silver-incorporated mesoporous calcium-silicate nanoparticles (Ag-MCSNs), resulting in lower cytotoxicity. The nanoparticles slowly released silver ions which could inhibit *Enterococcus faecalis (E. faecalis)* growth and colonization.^34^ CHX can be successfully loaded onto MCSNs by the mixing-coupling method. These nanoparticles released CHX, showing low cytotoxicity and excellent anti-*E. faecalis* properties. Moreover, they can also release Ca^2+^/SiO_3_^2−^ ions, showing an *in vitro* mineralization property. These nanoparticles could be developed into bone-defect-filling material or intracanal medications in dentistry.^35^

Huang, et al.^36^ (2017) loaded gentamicin and fibroblast growth factor 2 (FGF-2) with mesoporous calcium silicate nanoparticles, which could sustain the release of FGF-2 and gentamicin. These nanoparticles showed bone/cementum tissue regeneration and inhibited bacterial viability, suggesting their use in endodontics as a valuable, biocompatible dental pulp tissue regenerative material capable of odontogenesis. MCSNs can also be loaded with BMP-2 and prepared into a three-dimensional scaffold by 3D printing, inducing the differentiation of dental pulp cells and odontoblasts. The BMP-2-loaded mesoporous calcium silicate 3D scaffold could serve as an intracanal scaffold for both hemostatic clot retention and odontogenesis in reparative endodontic therapy.^21^ Nano-metal particles, such as Ag, Cu, copper oxide (CuO), and titanium oxide (TiO2), can serve as antibacterial agents in the oral cavity.^37,38^ In recent years, the toxic effects of metals and metal oxides have attracted significant attention.^39,40^ Thus, the safety of metal nanoparticles should be further investigated.^41^

Although many articles have highlighted the role of antimicrobials, there are still many disadvantages in using antibacterial drugs, including drug resistance, dysbiosis, etc. Therefore, active agents are needed to inhibit bacterial biofilms without affecting the ecological balance of flora, such as probiotics and prebiotics, thus reestablishing ecological balance or the biodiversity of oral microbiota.

### Organic nanoparticles

In recent years, nanoparticle carriers have commonly been prepared by the self-assembly of organic molecules, such as PLGA, chitosan, and poly (lactic acid).^42–45^

PLGA is a conventionally used component in preparing organic nanocarriers. In previous studies,^40,43^ PLGA-nanoparticles were prepared to encapsulate CHX. These nanoparticles could be delivered to the demineralized dentin matrix and the resin-dentin interface by dentinal tubules, in which they might play a role not only in inhibiting bacteria but also matrix metalloproteinase (MMP).^46^ PLGA is used to load grapeseed extract (GSE), which could improve the biodegradation resistance of demineralized dentin. Biodegradable polymer nanoparticles can deliver GSE via demineralized dentinal tubules, reducing the biodegradability of the dentin collagen matrix.^47^ PLGA is also used to load lovastatin and control its release, which can induce dentin differentiation at appropriate concentrations. Thus, these nanoparticles could serve as an adjunctive treatment in indirect pulp capping procedures.^48^ PLGA is used to deliver metronidazole or N-phenacylthiazolium bromide to modulate periodontitis progression.^49^ PLGA-chitosan nanoparticles are also loaded with simvastatin and doxycycline to promote the repair of infected periodontal sites and non-infected osseous defects.^50^ Compared with gel carriers, a PLGA particle carrier prolonged the release of active components, indicating its functional suitability for clinical applications.^51^

Liposomes are often used as drug carriers to encapsulate active ingredients.^52^ They were used to develop curcumin-loaded solid lipid nanoparticles (CurSLN) to treat oral mucosal infections, showing increased antibacterial activity.^53^ Furthermore, liposomes have served as carriers to prepare pH-responsive quaternary ammonium chitosan-liposome nanoparticles by first loading doxycycline with liposomes, then coating the latter with N, N, N-Trimethyl chitosan (TMC). When pH is low, the free amino group in the quaternary ammonium chitosan on the surface of nanoparticles will be protonated. The positive charge on the TMC surface could be increased to destabilize the nanoparticles, thereby triggering DOX release. These nanoparticles could be closely linked to biofilms and effectively destroy their membrane structure and disrupt bacterial biofilms *in vivo*.^54^

Chitosan is usually used as a carrier to load active ingredients. Chitosan nanoparticles loaded with toothpaste actives such as sodium fluoride (NaF) and cetylpyridinium chloride (CPC) are prepared by emulsion dispersion or ionic gelation. These nanoparticles continuously released their active ingredients, which makes them highly promising as dental delivery systems in the protection against caries.^55,56^ After functionalization with glutathione, chitosan showed a stronger ability to bind to chlorhexidine, resulting in a better release of chlorhexidine within 48 h.^57^ Moreover, polycaprolactone has been used to encase chlorhexidine to form Poly (ε-caprolactone) (PCL)-coated CHX nanocapsules. In this process, the nanocapsules could reach the demineralized dentin region via micron-scale dentin tubules, slowly releasing chlorhexidine, thereby playing an antibacterial role.^58^

Furthermore, chitosan has been used as a carrier to load dexamethasone (DEX), and alginate solution, containing transforming growth factor-β1(TGF-β1), to wrap the resulting nanoparticles. In turn, the core-shell nanosystem (TD-NS) could release TGF-β1 and DEX so materials might treat periapical inflammation.^59^ Zhu, et al.^60^ (2018) used the phosphorylated poly (amide-amine) dendrimer (PAMMA) as a carrier to load apigenin, a type of antibiotic insoluble in water. The material can both play an antibacterial role and promote remineralization. Polylactic acid (PLA) and PLGA have been used to load cells and BMP-2, respectively, promoting odontogenic differentiation and the formation of dentoid tissue via human stem cells of apical papilla (SCAP). The data showed great promise in promoting dentin tissue regeneration.^61^

Moreover, continuous release systems for CHX were developed based on chitosan (CS) and montmorillonite (MMT). The CHX-hybrid nanosystem containing positively-charged chitosan showed good mucoadhesion properties which enabled the drug delivery system to stay longer at the absorption site and, therefore, contributed to a beneficial effect on drug bioavailability.^62^ A pH-responsive and redox-sensitive polymer-based AmB-delivery carrier system was developed based on O-Carboxymethyl chitosan (OCMCH). This system was functionalized by conjugation with the antifungal peptide histatin 5, which targeted ligands and synergistic antifungal molecules against *Candida albicans*.^63^ The cellulose acetate phthalate nanoparticle is used to load chlorhexidine, enabling much greater infiltration into the subgingival tissue.^64^ Diblock copolymers underwent ultrasound treatment to form nanoparticle carriers by self-assembly. The recent nanoparticle carrier (NPC) DDS is flexible enough to co-load farnesol and myricetin. Therefore, nanoparticles could reduce *S. mutans*biofilm organization and acid production.^65^ Some inorganic nanoparticles are also used in oral mucosal diseases, such as hydroxyapatite nanoparticles and hyperbranched core-multishell nanocarriers.^66,67^

## Hydrogels

Hydrogels composed a natural or synthetic 3D network by physical or chemical strategies and could absorb a large amount of water (up to 1000-fold compared to the dry weight). Hydrogels consist of long polymer chains filled with water molecules between them.^68,69,70^ Natural hydrogels are biocompatible and can degrade into nontoxic by-products which can interact with biological macromolecules. However, their limitations lie in their weak mechanical strength and immunogenicity.^71^ By contrast, synthesized hydrogels can be better regulated and have better stability, but they might degrade into toxic by-products.^72^ Hydrogels can control drug release due to changes (swelling, dissolution or degradation) in the gel structure in response to internal or external stimuli.^73^ With appropriate release mechanisms, active drugs can be maintained locally at high concentrations for a longer period. Currently, the clinical applications of hydrogels for drug delivery include ophthalmology, cardiovascular diseases, and cancer, having attracted the attention of oral medicine in recent years.^74,75,76,77^ Compared to nanopore carriers, hydrogel carriers can maintain or trigger drug delivery and enable multi-drug delivery. At the same time, hydrogels avoid intravenous injection and have better local application prospects, making them interesting platforms for drug delivery in the oral cavity.^78^

### Chitosan hydrogels

In recent years, chitosan/β-glycerophosphate (CS/β-GP) thermosensitive hydrogels have served as drug delivery systems to load bone morphogenetic protein-7 (BMP-7) and ornidazole (ORN), continuously releasing them to treat periodontal diseases.^79^ Moreover, chitosan/β-glycerophosphate (CS/β-GP) thermosensitive hydrogels were loaded with aspirin to control inflammation and erythropoietin. Hydrogels can promote periodontal regeneration.^80^ They are also used to load naringin, a naturally derived polymethoxylated flavonoid compound with anti-inflammatory properties that inhibit the inflammatory state of periodontitis.^81^ Regarding other types of thermosensitive hydrogels, a thermosensitive micellar hydrogel was prepared from amphiphilic copolymer poly (ε-caprolactone-co-1,4,8-trioxa [4.6]spiro-9-undecanone)-poly(ethylene glycol)-poly (ε-caprolactone-co-1,4,8-trioxa [4.6]spiro-9-undecanone) (PECT) nanoparticles. Ibuprofen and basic fibroblast growth factor (bFGF) were encapsulated in these thermosensitive micellar hydrogels. Thus, these materials can promote anti-inflammatory activity and soft tissue healing.^82^

### Other hydrogels

There are other components of hydrogels. To promote tissue regeneration, biocompatible hydrogelator Nap-Phe-Phe-Tyr-OH (NapFFY) hydrogels were loaded with stromal cell-derived factor-1 (SDF-1) and bone morphogenetic proteins (BMPs) to promote and accelerate periodontal bone regeneration.^83^ Pan, et al.^84^ (2019) used PLGA–PEG–PLGA triblock copolymers as a carrier to load human periodontal ligament stem cells, which had been modified to overexpress platelet-derived growth factor-BB. These cells are capable of mediating enhanced alveolar bone regeneration* in vivo.* High-stiffness transglutaminase crosslinked gelatins (TG-gels) were developed, incorporated with IL-4 and stromal cell-derived factor (SDF)-1α, and used for periodontal tissue regeneration. The presence of IL-4 might promote M2 polarization of macrophages (Mφs) and osteogenesis in bone marrow-derived stromal cells (BMSCs).^85^ In the field of dental pulp regeneration, hyaluronic acid (HA) hydrogels were reinforced with cellulose nanocrystals (CNCs) and then, enriched with platelet lysate (PL) and GFs. The PL-loaded hydrogels showed preferential supportive properties for encapsulated human dental pulp cells (hDPCs) in *in vitro* culture conditions. These hydrogels could serve as scaffold for GF delivery and cell recruitment, with great potential in future developments for regenerative dentistry.^86^ Moreover, antibiotics and nitric oxide (NO), releasing biomimetic nanomatrix gel, were synthesized by the self-assembly of peptide amphiphiles, which could promote tooth revascularization with the maturation of root canals.^87^ Hydrogels can also be used to treat fungal infections and deliver anesthetics. Hydroxypropyl methylcellulose (HPMC) was used to load Histatin-5 (Hst-5), an antimicrobial peptide, which could treat oral candidiasis.^88^ A nanostructured lipid-biopolymer hydrogel was developed to continuously deliver lidocaine-prilocaine for trans-buccal preanesthesia, which showed stability (for 6 months in critical conditions) and suitable mechanical properties for oral administration.^89^ Natural products can also be used to make thermosensitive hydrogels for oral applications.^90^

## Nanofibers

Nanofibers are commonly produced from ceramic materials, metallic compounds, and synthetic polymers by electrospinning, phase separation, self-assembly or laser spinning.^91^ Nanofibers are defined as fibers approximately 100 nm in diameter, which can carry diverse antimicrobial molecules, thereby showing biocompatibility and a porous structure.^91,92^ High surface area and porosity are the main advantages of nanofibers. Drug release can be regulated by changing the porosity of nanofibers. Porous nanofibers can promote cell adhesion and shorten the drug release cycle, which is conducive to wound healing.^93^ In the oral cavity, nanofibers have structural characteristics similar to dental pulp and periodontal tissues when compared with the most prevalent nanoparticles. Moreover, nanofibers have a larger contact surface which helps distant delivery.^91^ Their advantage of easy modulation of drug release profiles depends on the properties of polymer/polymeric blends/other materials used.^94^ To treat *Actinomyces naeslundii*, *P. gingivalis* or *Enterococcus faecalis* infections, polydioxanone nanofibers were used to load antibiotics, such as metronidazole (MET), ciprofloxacin (CIP), and minocycline (MINO). The use of nanofibers suggested a significant potential in the eradication/elimination of bacterial biofilms.^95,96,97^ Moreover, a type of polydioxanone nanofiber was developed to load vascular endothelial growth factor (VEGF), which might be a promising scaffold for additional optimization in endodontic regenerative procedures.^98^

Moreover, polyvinyl alcohol, polylactic acid, and some natural products are also used to prepare nanofiber carriers. Thiolated chitosan (CS-SH) blended with polyvinyl alcohol (PVA) was used as the carrier to load α-mangostin, an antibacterial substance, and prepared into nanofibers. These nontoxic nanofibers could rapidly adhere to the buccal mucosa in the oral cavity, in which α-mangostin was released to inhibit oral bacterial flora and promisingly prevent the formation of dental caries.^99^ Resorbable PLA fibers based on electrospinning were developed, containing the antibiotic metronidazole. These fibers could slowly release metronidazole for the topical treatment of periodontitis.^100^ Cellulose nanofibers (CNF) were modified with κ-carrageenan oligosaccharides (CO) to deliver drugs, load surfactin, and manufacture surfactin-loaded CO-CNF. The surface-loaded CO-CNF showed antioxidant activity and inhibited the growth of *S. mutans*and *P. gingivalis* by preventing biofilm formation and reducing metabolic activity, which promoted oxidative stress in a concentration-dependent manner.^101^ However, most polymers currently used in nanofibers are expensive and not suitable for patients who have financial difficulties. At the same time, the stability of nanofibers and hosts’ immune responses should be further evaluated.^91^

## Films

In general, oral films serve as carriers of antibacterial or antimycotic agents for gradual and direct release at the target area in the oral cavity, which might be suitable for long-term local effects and improve therapy effectiveness.^102–106^ Mucoadhesive oral films (MOF) were prepared from carmellose by incorporating a nanotechnologically modified clay mineral intercalated with antiseptic drugs (chlorhexidine diacetate and digluconate) which could inhibitthe growth of *Staphylococcus spp.* and* Candida spp.*^107^ Gelatin films, as carriers to load econazole nitrate (ECN), were used as an imidazole antifungal agent to treat skin infections and mucosal candidiasis.^108^ The plasma treatment completely dissolved the gelatin within 48 hours in a simulated saliva solution, which suggested a potential application for gelatin films as the buccal delivery of econazole to treat oral candidiasis.^97^ Different hybrid nanofilms, composed of biopolymer matrices (chitosan, pectin, and chitosanpectin), were prepared and blended with nanostructured lipid carriers (NLC) and loaded in a eutectic mixture of 5% lidocaine-prilocaine (LDC–PLC). These nanohybrid films prolonged LDC-PLC release profile for more than 8 h* in vitro*. LDC-PLC shows higher drug permeation values across porcine oral mucosa and longer-lasting anesthesia without compromising its safety profile, indicating its significant potential to deliver local anesthetics.^22^

### Other Drug-delivery Systems

In dental tissue engineering, growth factors and scaffolds are widely used to provide a 3D-scaffold structure with a highly porous, interconnected network that enables the transport of cellular nutrients.^109–111^ Polycaprolactone (PCL)/polylactic-co-glycolic acid was combined with amorphous polycaprolactone (PCL) to synthesize scaffolds which were immobilized with adenoviruses onto the scaffold surface to locally deliver gene vectors encoding platelet-derived growth factor-BB or bone morphogenetic protein-7. Controlled scaffold microtopography, combined with localized growth factor gene delivery, improved the regeneration of periodontal bone–periodontal ligament (PDL) interfaces.^112^ Chitosan/alginate/PLGA hybrid scaffolds were prepared for control and the sequential delivery of competence factors, such as insulin-like growth factor (IGF-1), progression factor, and bone morphogenetic factor-6 (BMP-6), which induced proliferation and osteoblastic differentiation of cementoblasts for periodontal tissue regeneration.^109^

## Conclusion

Oral drug delivery systems have been designed in different ways to adapt to the physical and chemical environment of oral cavities for more suitable clinical applications. Carrier materials must have good biocompatibility and stable physical and chemical properties. The drugs loaded onto carriers are primarily antibacterial agents and bioactive substances that promote tissue growth. Temporary treatment with antibiotics is helpful in cases with severe symptoms of oral disease. Antibiotics can be used temporarily to suppress the dominant microbiota when severe dysbiosis occurs.

Indeed, we need to be alert to the abuse of antibiotics, which may lead to the dysbiosis of oral microbiota. Therefore, researchers need to pay attention to this issue when preparing carrier materials. Other drugs that preserve ecological balance may be considered for delivery in the future, such as prebiotic substrates.

Drug delivery systems can protect active substances in the complex oral environment and control their release to maintain an effective concentration. In general, hydrogels, scaffolds, and nanofibers can be used in tissue regeneration to treat periodontal and jaw defects because of their advantageous cell growth support. Carrier materials with good mucosal adhesion, such as films and hydrogels, can be used for mucosal drug delivery. Nanoparticles are often used to load active substances due to their strong dispersion, targeting bacteria or inflammatory cells. They have been widely used as drug delivery systems in the oral cavity to treat oral infectious diseases, such as caries and periodontal diseases. Therefore, effective carrier materials should continuously and steadily release effective drugs and target pathogenic bacteria, especially oral biofilms, to reduce cariogenic conditions without affecting the homeostasis of the oral flora and adapting to the oral environment.

Targeted and specific delivery systems may be the future development direction. For example, using the targeted delivery systems to adjust biofilm pH may prevent caries.

Researchers mainly use organic compounds to prepare carrier materials, but their biocompatibility and degraded byproducts need further verification. However, at present, drug delivery still faces challenges to its design and synthesis, and we lack a clear conclusion about the durability of its effect on resistant bacteria, hindering its clinical delivery. At the same time, there are few *in vivo* studies and animal experiments, and the biocompatibility of different drug delivery systems should be investigated further. The complexity of the oral environment and the lack of detailed studies on release rates and stability of specific drugs in drug delivery systems have become obstacles to their clinical application.
